# Dynamic phenotype monitoring to prevent genotype–phenotype discrepancies in pharmacogenetic-guided drug therapy

**DOI:** 10.3389/fphar.2026.1867629

**Published:** 2026-07-02

**Authors:** Pálma Porrogi

**Affiliations:** Faculty of Health and Sport Sciences, Széchenyi István University, Győr, Hungary

**Keywords:** biomarker matrix, dynamic phenotype monitoring, functional biomarker monitoring, pharmacogenomics, phenoconversion

## Abstract

**Background:**

One of the limiting factors for the clinical application of pharmacogenomics (PGx) is phenoconversion, i.e., the dynamic discrepancy between genotype-predicted and actual drug-metabolizing phenotypes. Systemic inflammation, polypharmacy, transporter dysfunction, redox and mitochondrial stress, and epigenetic modification can rapidly alter cytochrome P450 (CYP) enzyme and transporter activity. This suppression can lead to unexpected poor metabolizing phenotypes, increased drug accumulation, and even an increased risk of drug-induced liver injury. Therefore, static PGx genotyping should be complemented by real-time functional biomarker monitoring to achieve more accurate, effective, and safe drug delivery.

**Methods:**

This structured narrative review integrates a systematic literature search with expert-guided thematic synthesis. Mechanistic insights are supported by selectively included preclinical data. Candidate biomarkers—including miR-122, 4β-hydroxycholesterol, and GLDH—were identified through an iterative, criteria-based selection process that prioritizes mechanistic plausibility, clinical relevance, and favorable kinetics.

**Results:**

Mechanistic analyses have implicated cytokine-mediated signaling pathways (IL-6/STAT3, NF-κB), nuclear receptor repression (PXR/CAR), proteasomal CYP degradation, miRNA-driven mRNA destabilization, and enzyme inactivation as causes of phenoconversion. Dysfunction of the transporters OATP, BSEP, and MRP2, particularly in *SLCO* and *ABCC* genetic variants, creates a dual intrahepatic bottleneck that exacerbates drug and metabolite accumulation. The biomarker matrix, which collectively considers 4β-hydroxycholesterol, miR-122, GLDH, M30, sCD163, and acute phase reactants (CRP/IL-6), provides a theoretical framework to explore early hepatocellular stress secondary to inflammation-mediated CYP suppression and phenoconversion, thereby serving as an investigative tool to model genotype–phenotype discordance and anticipate potential variations in drug exposure tolerance.

**Conclusion:**

Within this hypothesis-generating framework, an integrated analysis of routine (CRP, transaminases), functional, and molecular biomarkers offers a novel strategy for interpreting clinically significant genotype–phenotype discordance. This approach shows the functional consequences of altered CYP activity rather than genetic predictions. Combining these multi-level parameters systematically reflects the main mechanistic domains: systemic inflammation-induced phenoconversion (CRP, IL-6), mitochondrial and oxidative stress (AST/ALT ratio, GLDH), early hepatocyte stress and apoptosis (miR-122, M30), and actual CYP3A4 metabolic capacity (4β-OHC). A PGx panel integrated with a dynamic biomarker could lead to safer, more effective, and adaptive drug dosing and reduced liver injury for high-risk patients.

## 1 Introduction

The complexity of human biology limits a static, exclusively genotype-based perspective. Cellular processes function as interconnected, non-linear networks that generate dynamic responses to internal and external stimuli over time. Drug metabolism and pharmacological response are influenced by more than germline genetic variants. Phenotype, including metabolic capacity and drug sensitivity, may fluctuate over time and across contexts due to environmental factors, inflammatory states, comorbidities, and drug–drug interactions (DDIs). Understanding this dynamic transformation, known as phenoconversion, is essential for ensuring the efficacy and safety of personalized pharmacotherapy (Shah and Smith, 2015a; Klomp et al., 2020).

The cytochrome P450 (CYP) enzyme family, particularly the CYP1A2, CYP2D6, CYP2C9, CYP2C19, and CYP3A4/5 isoforms, plays a central role in drug biotransformation (Zanger and Schwab, 2013). Genetic polymorphisms significantly influence these enzymes’ activity, but their expression and function are also modulated by acquired factors throughout life. These include acute and chronic inflammation, changes in hepatic and renal function, environmental exposures such as smoking and diet, and, notably, polypharmacy. During systemic inflammation, pro-inflammatory cytokines—primarily interleukin-6 (IL-6), tumor necrosis factor-α (TNF-α), and interleukin-1β (IL-1β)—reduce CYP expression and activity via transcriptional and post-translational mechanisms. These processes partly involve the suppression of nuclear receptors such as the pregnane X receptor (PXR) and the constitutive androstane receptor (CAR), but may also involve changes in mRNA stability, protein degradation, or post-translational modifications (Schmith and Foss, 2008; Shah and Smith, 2015b). Cytokine-mediated inhibition is rapid and generally reversible, suggesting that metabolizer status predicted by genotype may change within a short timeframe (Schmith and Foss, 2008; Huntjens et al., 2010; Shah and Smith, 2015b).

The clinical consequences of these phenotypic modifications are well established. For instance, acute inflammation can reduce CYP2C19 activity, decreasing the formation of clopidogrel’s active metabolite and increasing thrombotic risk (Klomp et al., 2020). Inhibition or functional reduction of CYP2D6 impairs the conversion of codeine to morphine, resulting in reduced analgesic efficacy. Additionally, elevated IL-6 levels are associated with decreased CYP3A4 activity, which can increase tacrolimus or certain statin concentrations, thereby increasing the risk of toxicity (Shah and Smith, 2015b; Klomp et al., 2020). These examples demonstrate that phenoconversion has direct and significant implications for therapeutic efficacy, clinical decision-making, and patient safety.

To prevent overgeneralization of phenoconversion, it is essential to distinguish several related but distinct processes along the pharmacogenomics (PGx)–exposure–toxicity axis. In this review, pharmacogenomic phenoconversion is defined narrowly as those cases in which the observed activity is of phase I. CYP enzymes differ from those predicted by the germline genotype. For example, a normal CYP2D6 metabolizer may exhibit poor metabolizer function under strong inhibition. This phenomenon is conceptually distinct from disease-related suppression of drug metabolism, such as inflammation-mediated regulation of CYP expression, which can occur independently of genotype. Furthermore, biomarkers of drug-induced liver injury (DILI), such as miR-122, GLDH, and M30, primarily indicate hepatocyte stress or injury. They should not be considered direct measures of CYP phenotype, even though injury and impaired metabolism may coexist with phenoconversion. Alternatively, transporter-mediated changes in drug exposure, such as those via OATP1B1, BSEP, or MRP2, may significantly affect intrahepatic and systemic drug concentrations. Accordingly, this manuscript distinguishes among (1) metabolic phenotype (CYP/transporter function), (2) hepatotoxicity risk and hepatocellular injury, and (3) clinical drug exposure. It examines how inflammation, polypharmacy, and genetic variation interact across these overlapping yet distinct levels. This framework acknowledges that no single factor universally influences all elements of PGx, but that each contributes specifically and proportionally to different aspects of drug response.

Despite recent advances, several unresolved questions persist in the scientific literature. These include integrating time-dependent cytokine profiles (e.g., IL-6), enzyme activity measurements, drug concentrations, and clinical outcomes with genotype data. There is also a need to elucidate causal mechanisms between CYP metabolism and inflammation, quantitatively assess the role of drug metabolites in phenoconversion induction, and validate algorithms that integrate real-time biomarkers with therapeutic drug monitoring (TDM) data for real-time, dynamic response monitoring and dose modification in PGx-based drug therapy for efficacy and safety. Biomarker monitoring, especially integrating cytokine profiles like IL-6, functional phenotype measurements, and time-dependent drug concentrations, is essential to bridge the genotype–phenotype gap and accurately predict metabolic responses. Multi-omic, multi-level integrated approaches enable real-time detection and quantification of inflammation- or drug metabolite–induced phenoconversion. This is critical for proactive dose adjustments, minimizing adverse effects, and maximizing therapeutic efficacy. Additionally, analytical validation of biomarkers, establishing reference ranges for various clinical contexts, and evaluating the cost-effectiveness and clinical applicability of monitoring are necessary.

In summary, CYP enzyme system activity is regulated in multiple dimensions, and phenoconversion can supersede genotype-predicted metabolizer status in diverse clinical scenarios. The modifying effects of inflammatory cytokines such as IL-6, TNF-α, and IL-1β, as well as certain drug metabolites, produce dynamic, context-dependent pharmacokinetic changes (Schmith and Foss, 2008; Drozdzik et al., 2019; Li et al., 2019). Achieving personalized and safe pharmacotherapy requires prospective, integrated studies and standardized biomarker monitoring (Church and Watkins, 2018; Li et al., 2019). This review aims to: (i) summarize current and emerging biomarkers relevant to pharmacogenomic phenoconversion and drug metabolism changes; (ii) explain how these biomarkers relate to metabolic phenotype, hepatotoxicity risk, and drug exposure; (iii) identify key research gaps; and (iv) propose real-time biomarker-based monitoring strategies that assess phenotype in relation to genotype, as indicated by PGx testing. These strategies will integrate biochemical, metabolomic, and pharmacological data to improve efficacy and reduce toxicity, including hepatotoxicity, in individualized drug therapy (Klomp et al., 2020; Ali and Mahato, 2026).

## 2 Scope and aims

The manuscript is a hypothesis-driven, structured narrative review that outlines and justifies the need for dynamic precision-based monitoring and offers a conceptual framework for pharmacotherapeutic monitoring to enhance drug response and safety outcomes (Church and Watkins, 2017; 2018; Marsousi et al., 2017; Ali and Mahato, 2026). While genotype-based predictive approaches are valuable, they are insufficient on their own. Combining static genomic information with temporally resolved phenotypic biomarkers may help to effectively mitigate adverse effects, toxicity, and DILI resulting from phenoconversion (Qian et al., 2024; Yang et al., 2025).

Genotype information is a critical starting point for individualized pharmacotherapy; however, it is inherently static and has limitations. Phenoconversion dynamically influences the activity of CYP enzymes and other key drug-metabolizing enzymes, as well as overall drug metabolism. Therefore, predictive accuracy requires supplementing and integrating multidimensional, multi-omic, and time-dependent data.

Within this hypothesis, three overarching questions are addressed in this review:

1. Which mechanistic pathways, including inflammation, redox stress, and transporter–enzyme interactions, plausibly drive divergence between genotype-predicted and observed metabolic phenotypes?
2. Which biomarkers—routine, functional, and molecular—have sufficient mechanistic rationale and emerging evidence to estimate the risk of phenoconversion?
3. How can these markers be conceptually integrated into patient population–specific frameworks to improve understanding and prediction of phenoconversion risk?

## 3 Materials and methods

### 3.1 Study design

This manuscript is a structured narrative review. The primary aim is to synthesize mechanistic, translational, and clinical evidence on inflammation- and drug-induced phenoconversion and to propose a biomarker-guided conceptual framework for clinical PGx. The review does not claim to be an exhaustive systematic review or a quantitative meta-analysis; instead, it combines a systematic search strategy with expert-guided thematic synthesis.

### 3.2 Eligibility criteria and study selection

The literature search and study selection adhered to PRISMA 2020 principles and were designed to be systematic yet hypothesis-based rather than fully exhaustive. Initial database searches identified 620 records, of which 175 duplicates were removed before screening. A total records were then screened at the title–abstract level for relevance to at least one of the following domains: (i) molecular mechanisms of phenoconversion; (ii) clinical evidence of genotype–phenotype discordance (iii) evaluation of candidate biomarkers (e.g., miR-122, GLDH, 4β-hydroxycholesterol, M30, sCD163, CRP/IL-6); in the context of DILI, inflammation, or CYP3A4 function; and (iv) clinical monitoring and dosing strategies in high-risk populations. Based on these criteria, 315 records were excluded (Supplementary Material 1).

Full texts were sought for 130 articles; six could not be retrieved (e.g., due to unavailability or lack of author response), leaving 124 studies for detailed eligibility assessment. At this stage, exclusion criteria comprised: inappropriate study type or population (e.g., reviews, meta-analyses, protocols, or studies not involving a relevant clinical population; n = 35), review/meta-analysis only without primary data suitable for extraction (n = 25), and insufficient methodological or quantitative detail to support meaningful synthesis (n = 10). Human clinical studies (trials, cohort and case–control studies, and large case series) served as the primary evidentiary basis for clinical interpretations and hypothesis-generating proposals. Preclinical *in vivo* and *in vitro* studies were retained selectively when they provided unique mechanistic insight—such as cytokine-mediated regulation, mitochondrial dysfunction, transporter internalization, or detailed signaling interactions—that could not be inferred from human data alone.

In total, 54 studies met the inclusion criteria and were incorporated into the narrative review. Inclusion criteria were: (i) human studies as the main source of clinical inference; (ii) evaluation of pharmacogenetic/pharmacogenomic and/or other omic (transcriptomic, proteomic, metabolomic, epigenomic, microbiome) biomarkers in relation to a pharmacokinetic or pharmacodynamic drug-response phenotype; (iii) quantitatively or clearly interpretable reporting of biomarker–phenotype relationships; and (iv) explicit relevance to phenoconversion and/or pharmacogenomics-guided multi-omic precision pharmacotherapy.

### 3.3 Sources of information and search strategy

A structured literature search was performed in PubMed/MEDLINE, Embase, Web of Science, and Google Scholar for articles published from January 2000 to March 2026. The search strategy used both Medical Subject Headings (MeSH) and free-text terms relevant to the core concepts of this review, such as *pharmacogenomics*, *phenoconversion*, *cytochrome P450* (CYP), *inflammation*, *IL-6*, *biomarker*, *miR-122, 4β-hydroxycholesterol, GLDH, M30, drug-induced liver injury (DILI)*, *transporters* (*SLCO1B1*, *ABCB1*, *ABCC2*), and *therapeutic drug monitoring*. Boolean operators (AND, OR) combined search terms. An example PubMed search string is in the Supplementary Material. Additional references were identified by screening reference lists of key original articles and reviews.

### 3.4 Quality considerations and weighting of evidence

Although no formal risk-of-bias scoring was applied to all included studies, methodological quality was explicitly considered. For clinical studies, priority was given to prospective designs, well-characterized populations, clearly defined endpoints, and appropriate analytical methods. For preclinical work, attention focused on model relevance (species, cell type), concentration ranges, controls, and internal consistency. In the narrative synthesis, greater interpretive weight was given to findings from higher-quality human studies. Preclinical data were used primarily to support mechanistic plausibility rather than to define clinical cut-offs.

### 3.5 Rationale for biomarker selection

The biomarker panel discussed in this review was assembled through an iterative, criteria-based process during the literature review. Candidate biomarkers were retained if they (i) had a clear mechanistic link to key pathways of interest, such as miR-122 as a hepatocyte-specific stress marker, 4β-OHC as an endogenous CYP3A4 activity marker, or GLDH as a mitochondrial injury marker; (ii) showed clinical association with phenoconversion, DILI, or related outcomes in at least one independent human study; (iii) demonstrated favorable temporal kinetics, with early changes preceding standard liver tests; and (iv) were measurable using established analytical methods such as qPCR, LC-MS/MS, or ELISA, with potential for clinical implementation. These criteria ensured that the final biomarker matrix integrates mechanistically relevant, clinically anchored, and technically feasible markers.

## 4 Results

### 4.1 Conceptual distinctions: metabolic phenotype, hepatic injury, and exposure

Multiple, partially overlapping processes influence drug response and may mimic or alter pharmacogenomic predictions of phenotypes. Metabolic phenotype primarily reflects the functional activity of CYP enzymes and hepatic transporters, measured using probe drugs, endogenous markers, or changes in drug exposure. This concept is distinct from hepatocellular injury and DILI, which are identified by injury biomarkers such as miR-122, GLDH, and M30, as well as by histopathological findings. Clinical drug exposure results from the cumulative effects of metabolism, transport, protein binding, and organ function.

Inflammation-mediated CYP suppression, polypharmacy-related enzyme inhibition, mitochondrial injury, and transporter dysfunction often co-occur but do not follow a single linear pathway. In some patients, inflammation primarily affects CYP activity without causing overt injury. In others, mitochondrial or cholestatic mechanisms may be more prominent, resulting in only modest changes in CYP phenotype. Meaningful interpretation requires distinguishing metabolic capacity, injury risk, and drug exposure before evaluating their interactions in specific clinical contexts.

### 4.2 Inflammation-mediated modulation of CYPs and transporters

#### 4.2.1 *In vitro* and preclinical evidence

*In vitro* studies consistently show that pro-inflammatory cytokines, including IL-6, TNF-α, and IL-1β, suppress the expression and activity of multiple CYP enzymes through transcriptional and post-translational mechanisms (Schmith and Foss, 2008; di Masi et al., 2009). These mechanisms include inhibition of nuclear receptors (PXR, CAR, RXRα), altered nuclear translocation, destabilization of CYP mRNA, and increased ubiquitin–proteasome-mediated degradation of CYP proteins (di Masi et al., 2009; Shah and Smith, 2015b). Similar cytokine-driven regulation occurs for uptake and efflux transporters, such as OATP1B1/1B3, BSEP, and MRP2. However, these effects are isoform-specific (Liptrott et al., 2009). Preclinical data support a model in which acute inflammatory signaling reduces the pool of functional CYP and transporter proteins, thereby decreasing intrinsic clearance and altering tissue distribution. The direction and magnitude of these changes vary by enzyme and transporter; for example, CYP1A2, CYP2C19, CYP2D6, CYP2C9, CYP3A4/5, UGTs, and various transporters show non-uniform responses across models (Jong et al., 2020; Lenoir et al., 2021).

#### 4.2.2 Human pharmacokinetic (PK) evidence

Human pharmacokinetic studies provide converging but heterogeneous evidence that systemic inflammation reduces the activity of several CYP isoforms. Clinical data show decreased apparent clearance or reduced metabolic ratios for substrates of CYP3A4, CYP2C19, CYP2C9, and CYP2D6 with elevated inflammatory markers (Schmith and Foss, 2008; Klomp et al., 2020; Lenoir et al., 2021; Yokota et al., 2025). In pediatric populations and in critical illness, IL-6 and CRP have consistently been linked to reduced CYP3A4/5 activity, as indicated by tacrolimus concentration–dose ratios and endogenous markers such as 4β-OHC (Diczfalusy et al., 2009; van der Wouden et al., 2022; Yokota et al., 2025).

Not all CYP enzymes are affected equally, nor do they follow the same temporal pattern. For example, CYP1A2 may be regulated differently from CYP3A4, and CYP2D6 appears less sensitive than CYP2C19 or CYP3A4 in some cohorts (Jong et al., 2020; Klomp et al., 2020). Although IL-6 concentrations in the low tens of pg/mL have been linked to measurable CYP3A4 suppression in some studies, these values should be considered context-dependent correlates rather than universally validated clinical thresholds.

#### 4.2.3 Clinical outcome evidence

In clinical settings, inflammation-related changes in enzyme activity appear as discrepancies between genotype-predicted and observed drug responses. Reduced CYP2C19 activity during acute inflammation is linked to impaired clopidogrel activation and increased thrombotic events (Klomp et al., 2020; Tornio et al., 2022). Similarly, decreased CYP3A4 activity in the presence of elevated IL-6 is associated with higher tacrolimus and statin concentrations, increasing the risk of nephrotoxicity, myotoxicity, and other dose-related adverse effects.

These examples demonstrate that inflammation-mediated phenoconversion is both context- and pathway-specific. The extent of phenoconversion depends on which CYP enzymes or transporters govern a particular drug’s disposition, the degree of their downregulation in the disease state, and the presence of additional injury or transporter defects that may further alter drug exposure.

### 4.3 Polypharmacy, DDI, and mechanism-based inactivation

#### 4.3.1 Mechanistic and preclinical evidence

*In vitro* and preclinical studies show that co-administered drugs may act as reversible competitive inhibitors or mechanism-based inactivators of CYP enzymes, leading to a time-dependent decline in functional enzyme activity (Fahmi et al., 2008; Concha et al., 2023; Stingl and Viviani, 2025). Lipophilic parent drugs and their metabolites can accumulate in hepatocytes, saturate metabolic pathways, and generate reactive intermediates that covalently modify CYP heme centers or active sites, thereby prolonging enzyme dysfunction.

These processes are quantified by kinetic parameters such as Km, v_max_, K_i_, IC_50_, and kinact, which determine the potency and timing of inhibition or inactivation (Church et al., 2025). Although usually obtained from *in vitro* systems, these parameters provide a mechanistic framework for understanding how complex multidrug regimens can alter metabolic capacity beyond genotype-predicted expectations.

#### 4.3.2 Human PK evidence

Clinical PK studies show that DDI can significantly alter CYP substrate levels, affecting the functional phenotype. For example, methotrexate (MTX) is a folate antagonist widely used in oncology and in the treatment of autoimmune disorders. Unlike many drugs susceptible to phenoconversion via CYP modulation, MTX metabolism is not primarily CYP-dependent. Instead, MTX is predominantly eliminated unchanged via renal excretion, primarily through glomerular filtration and active tubular secretion, with minor hepatic metabolism via aldehyde oxidase and minimal involvement of phase I/II CYP enzymes (. Key renal transporters involved in MTX clearance include the organic anion transporters (OAT1/3, SLC22A6/8) and multidrug resistance–associated proteins (MRP2/4, ABCC2/4) (Łapczuk-Romańska et al., 2023).

Toxicity concerns with MTX center on delayed elimination, most often attributable to impaired renal function or transporter-mediated DDIs. Conditions that reduce renal perfusion, glomerular filtration rate, or transporter activity (e.g., acute kidney injury, dehydration, co-administered nephrotoxic agents) place patients at risk for MTX accumulation and subsequent hepatotoxicity, myelosuppression, and mucositis (Howard et al., 2016). Therefore, in the context of phenoconversion, the focus shifts from CYP modulation to renal and transporter function as the principal determinants of MTX clearance and toxicity risk.

#### 4.3.3 Clinical outcome evidence

Clinically, polypharmacy-related interactions lead to higher rates of toxicity or therapeutic failure. Combination chemotherapy regimens, such as cyclophosphamide with docetaxel, are linked to increased hepatotoxicity compared with monotherapy (Bezabeh et al., 2012; Chioukh et al., 2014). The interaction between MTX and PPIs is well documented and is not mediated by CYP inhibition. Instead, PPIs such as omeprazole, esomeprazole, and pantoprazole can delay the renal elimination of MTX, leading to increased systemic exposure and toxicity (Chioukh et al., 2014). Mechanistically, this DDI is attributed to inhibition of renal tubular transporters—specifically MRP2 (ABCC2) and breast cancer resistance protein (BCRP, ABCG2)—which are responsible for the tubular secretion of MTX and its metabolites (Chioukh et al., 2014; Drozdzik et al., 2019; Wang et al., 2020). By inhibiting these transporters, PPIs reduce MTX excretion and extend its half-life, especially in high-dose therapy settings.

Thus, the clinically relevant risk is not CYP-mediated phenoconversion but rather transporter-mediated accumulation. Recognition of this mechanism is crucial for avoiding potentially life-threatening MTX toxicity. Current guidelines recommend withholding PPIs during high-dose MTX therapy and closely monitoring renal function and plasma MTX concentrations in any patient requiring concomitant PPI use (Howard et al., 2016; Wang et al., 2020).

### 4.4 Chronic inflammation, epigenetic remodeling, and persistent phenotype shifts

Chronic low-grade inflammation influences hepatic drug metabolism through mechanisms that partially overlap with, but differ from, those during acute cytokine surges. In the acute-phase response, decreased albumin and increased α1-acid glycoprotein levels alter the pharmacologically active fraction of drugs, thereby modifying unbound clearance and potentially increasing efficacy or toxicity, especially for highly protein-bound drugs with a narrow therapeutic index (Adipose Tissue, Inflammation, and Cardiovascular Disease | Circulation Research, n. d.). These protein-binding changes are primarily distributional and do not cause phenoconversion unless sustained changes in enzyme or transporter function occur.

In contrast, chronic low-grade inflammation leads to persistent reprogramming of transcriptional and epigenetic processes in hepatocytes. Prolonged exposure to mediators such as TGF-β, IL-6, and other cytokines activates DNA methyltransferases and histone-modifying enzymes, resulting in hypermethylation of CYP gene promoters, histone deacetylation, and recruitment of repressor complexes (Shah and Smith, 2015b; Church et al., 2025). Chronic inflammation also induces sustained changes in miRNA profiles, including miR-122 and miR-192, which further suppress CYP expression (Shah and Smith, 2015b; Zhang et al., 2024). Together, these molecular changes limit mRNA synthesis and translation, reduce levels of phase I/II enzymes and transporters (OATP, BSEP, MRP2), and weaken hepatic metabolic resilience while increasing drug sensitivity (Evers et al., 2018).

As a result, in chronic liver disease, metabolic syndrome, or the tumor microenvironment, PGx predictions of rapid metabolizer phenotypes may not be observed (Table 1). Prodrugs like clopidogrel or some chemotherapeutic agents may be inadequately activated and fail to reach therapeutic levels. Meanwhile, drugs with a narrow therapeutic index, such as tacrolimus or certain tricyclic antidepressants, may reach unexpectedly high levels, raising the risk of hepatotoxicity, nephrotoxicity, or myelosuppression. In these cases, the observed phenotype results from the combined effects of genotype, chronic inflammatory remodeling, organ dysfunction, and, often, polypharmacy, rather than a single factor.

**TABLE 1:** Comparative molecular architecture of acute vs. chronic inflammation-induced phenoconversion.

Cellular level	Acute systemic inflammation (cytokine storm/Sepsis)	Chronic latent inflammation (tumor microenvironment)	Predictive clinical biomarkers
Signal transduction	Precipitous activation of JAK/STAT3 and NF-κB pathways via IL-6 and TNF-a surges	Smoldering IL-1β and TGF-β signaling; persistent low-grade systemic inflammatory burden	Acute: IL-6 (>20 pg/mL), CRP (>30 mg/L). Chronic: sCD163, GlycA
Transcriptional control	Rapid silencing: Inhibition of PXR, CAR, and RXRa nuclear translocation; immediate halt of CYP mRNA synthesis	Epigenetic remodeling: Activation of DNA methyltransferases (DNMTs); hypermethylation of CYP promoter regions	Dynamic flux: Serum 4β-OHC.
Post-translational regulation	Accelerated ubiquitin-proteasome degradation of existing CYP proteins; marked mRNA instability	Shift in stable micRNA profiles (e.g., miR-122, miR-192); histone deacetylation and chromatin compaction	Liquid biopsy: Elevated exosomal miR-122 and miR-192 levels
Redox homeostasis	Acute GSH depletion; massive ROS/RNS production leading to immediate oxidative distress	Sustained oxidative stress; adaptive but “strained” antioxidant response; chronic lipid peroxidation	Redox status: GSH/GSSG ratio; malondialdehyde or isoprostanes
Mitochondrial bioenergetics	Mitochondrial catastrophe: mPTP opening, cytochrome-c release, and precipitous ATP collapse	Bioenergetics erosion: Progressive mitochondrial DNA (mtDNA) damage; impaired fatty acid β-oxidation	Mitochondrial stress: GLDH (glutamate dehydrogenase), de ritis ratio (AST/ALT >1.2)
Transport and kinetics	Functional cholestasis via BSEP and MRP2 internalization; rapid decline in serum albumin	Downregulation of uptake transporters (OATP1B1/1B3); interplay with hepatic steatosis	Functional output: Total bile acids; keratin-18 fragments (M30 for apoptosis)
Clinical phenotype	Phenoconversion: Sudden shift to “poor metabolizer” status within hours; high risk of acute toxicity	Metabolic resilience erosion: Gradual “silent DILI”; progressive loss of the therapeutic window over weeks/months	Integrated index: Genotype + IL-6 + miR-122 weighted risk score

Additional metabolic and immunological mechanisms amplify these effects. Cytokine-induced suppression of CYP expression and activity can cause metabolite accumulation and increased reliance on glutathione (GSH)-mediated detoxification (Church et al., 2020; Aubrecht et al., 2025). When GSH is depleted, electrophilic intermediates may form covalent adducts with proteins and lipids, triggering immune dysregulation, cellular stress, and mitochondrial dysfunction, including membrane depolarization and opening of the mitochondrial permeability transition pore (mPTP).

In summary, chronic inflammation does not uniformly induce phenoconversion across all CYP enzymes and transporters. Instead, it creates a background of reduced metabolic reserve and heightened vulnerability, within which genotype and acute triggers, such as infections or DDI, can more readily precipitate clinically significant phenotype shifts.

Phenoconversion may involve both acute and chronic components, each with distinct clinical implications. In the acute phase, cytokine-mediated CYP suppression occurs rapidly and is often reversible; clinicians should anticipate enzyme recovery, or re-phenoconversion, to avoid subtherapeutic drug levels as inflammation resolves. In the chronic phase, persistent deficits recover slowly due to structural and epigenetic alterations. Standard transaminase measurements may fail to detect early or latent dysfunction, whereas biomarkers such as IL-6, the De Ritis ratio, and GLDH offer greater sensitivity (Lu and Cederbaum, 2008; Thulin et al., 2017; Church et al., 2020; 2025). Therapeutic strategies should be tailored to the phase: acute inflammation requires temporary dose reduction or discontinuation of high-risk drugs. In contrast, chronic inflammation necessitates long-term dose adjustments and supportive measures to preserve residual enzymatic function.

### 4.5 Transporter-mediated changes in drug exposure and their relationship to phenoconversion

Hepatic uptake and efflux transporters are key determinants of intrahepatic and systemic drug concentrations and often act independently of CYP genotype. Transporters such as OATP1B1/1B3, OCT1, BSEP, MRP2, and OSTα/β regulate hepatocellular uptake and biliary excretion of many clinically significant drugs and metabolites (Liptrott et al., 2009; Drozdzik et al., 2019). The Km values for OATP1B1 substrates typically fall within the micromolar range. Inhibition or genetic reduction of OATP1B1 function can increase plasma concentrations of drugs, including certain statins. In contrast, BSEP inhibition may lead to intracellular bile acid accumulation and cholestasis.

In PGx, transporter-mediated changes in drug exposure are related to, yet distinct from, CYP phenoconversion. For instance, individuals carrying the SLCO1B1*5 allele and with normal CYP3A4 activity may still exhibit elevated statin exposure due to diminished hepatic uptake (Roszkiewicz et al., 2021; Seo and van der Graaf, 2022). Conversely, patients with functional transporters but cytokine-induced CYP3A4 suppression may experience increased concentrations of tacrolimus or other CYP3A4 substrates (Shah and Smith, 2015b; Ali and Mahato, 2026; Summary Annotation, 2026). When both mechanisms are present, such as an *SLCO1B1* risk allele combined with IL-6-mediated *CYP3A4* downregulation, a dual bottleneck occurs. This scenario involves reduced hepatic uptake or excretion and impaired metabolic clearance, substantially increasing the risk of toxicity even in individuals with otherwise favorable PGx profiles (Fahmi et al., 2008; Roszkiewicz et al., 2021).

Clinical evidence underscores the significance of transporter-mediated mechanisms in DILI and altered drug exposure. Polymorphisms in *OATP1B1* and *ABCC2* have been linked to MTX toxicity, while downregulation of BSEP and MRP2 has been associated with functional cholestasis and hyperbilirubinemia in patients treated with hepatotoxic drugs (Pichini et al., 2023; Church et al., 2025). In these scenarios, transporter dysfunction alters drug exposure and toxicity risk independently of the metabolic capacity predicted by CYP genotype. Thus, a comprehensive approach to predicting drug toxicity should include both genetic and functional assessments of transporters in addition to conventional metabolic profiling.

### 4.6 Integrative perspective: overlapping yet distinct pathways

The evidence suggests that CYP suppression via inflammation, metabolite accumulation, mitochondrial injury, liver damage, and transporter dysfunction form overlapping, context-dependent networks rather than distinct, isolated phenoconversion pathways (Zhou et al., 2009; Hoofnagle and Björnsson, 2019; Jong et al., 2020; Thakur et al., 2024; Church et al., 2025). In some clinical scenarios, inflammation-driven CYP downregulation primarily determines phenotype. In others, transporter polymorphisms, mitochondrial susceptibility, or DDIs have greater influence. The concept of pharmacogenomic phenoconversion applies best when genotype-predicted and observed CYP or transporter function diverge. Redox stress, hepatocellular injury, and DILI biomarkers should be viewed as modifiers of vulnerability and harm (Jaeschke et al., 2012; 2012; Hoofnagle and Björnsson, 2019; Pichini et al., 2023; Thakur et al., 2024; Yang et al., 2025; Yokota et al., 2025). Exposure metrics represent the integrated outcome of these interconnected processes.

This mechanistic stratification serves as the basis for the hypothesis-generating framework of this review (Toda et al., 2021; Luo et al., 2023). Dynamic, multi-domain biomarker monitoring is promising because distinct constellations of pathways contribute to risk in different patients and clinical contexts, rather than a unified cascade of processes.

### 4.7 Biomarkers: Rationale, selection, and clinical integration

Bridging the diagnostic gap between genotype-based predictions and real-time clinical outcomes requires supplementing traditional laboratory parameters, such as CRP, transaminases, bilirubin, and ALP, with modern molecular and functional indicators. A dynamic, multi-domain biomarker framework may enable earlier detection of cellular alterations, improve recognition of pharmacogenomic phenoconversion, and support risk-adapted therapeutic decisions. The panel includes liver-specific, mitochondrial, apoptotic, immunometabolic, and functional CYP markers. In keeping with the hypothesis-generating nature of this review, these biomarkers are not proposed as a validated, prescriptive score but as components of a conceptual matrix that can inform the design of future validation studies aligned with our three core hypotheses (mechanistic drivers of phenotype shifts, biomarker-based detection, and precision monitoring).

#### 4.7.1 Selected biomarkers

**MiR-122** is the most abundant miRNA in hepatocytes among liver-specific biomarkers (Antoine and Dear, 2017; Zhang et al., 2024). It regulates lipid and stress responses under physiological and pathological conditions. During cellular injury, miR-122 is rapidly released into circulation (Thulin et al., 2017; Zhang et al., 2024). Clinically, elevations in miR-122 often precede increases in ALT or AST and strongly correlate with hepatocyte stress and injury, making it an ideal early indicator for subclinical DILI and phenoconversion (Lee et al., 2017; Thulin et al., 2017). Analytical quantification requires PCR-based techniques. Interpretation must consider preanalytical variability, including hemolysis and sample handling. Currently, miR-122 assays are not widely available in all clinical laboratories and are typically offered through specialized centers or research facilities. Turnaround times range from several days to a week. These constraints highlight that, at present, miR-122 is better suited as a research tool and early-signal biomarker in specialized settings than as a universally available clinical test.

The endogenous metabolite **4β-OHC** is a marker of CYP3A4-mediated oxidative capacity (Fahmi et al., 2008; Diczfalusy et al., 2009). Unlike probe-substrate-based phenotyping, 4β-OHC exhibits slower serum kinetics, providing a more integrative and long-term indicator of CYP3A4 function. In patients receiving tacrolimus or certain statins, decreased 4β-OHC levels indicate CYP3A4 suppression and reduced metabolic capacity, while increased levels reflect enzyme induction (Dutreix et al., 2014; Li et al., 2019). CYP3A4 genotype (e.g., CYP3A4 *1/1) alone is not a robust or actionable pharmacogenomic phenotype, in contrast to CYP2D6 or CYP2C19 phenotypes referenced in clinical guidelines. Thus, 4β-OHC and drug concentrations serve as relevant functional measures of CYP3A4 activity to detect phenoconversion. Concurrently, other novel parameters serve as sensitive indicators of subcellular damage; specifically, GLDH is an early marker of mitochondrial integrity loss (Church et al., 2020; Aubrecht et al., 2025). Elevation of GLDH is particularly informative when apoptosis markers such as M30 are absent, as GLDH specifically indicates mitochondrial matrix disruption rather than simple, transient disruption of hepatocyte membrane integrity. Because of its high molecular weight, GLDH leakage requires structural organelle breakdown, making it especially valuable in characterizing underlying pathways in cases of acetaminophen overdose, valproate toxicity, or mechanism-based inactivation (Thulin et al., 2017; Aubrecht et al., 2025).

The **M30 peptide**, a cleaved keratin-18 fragment, is a specific marker of caspase-mediated apoptosis (Thulin et al., 2014; Korver et al., 2021). Rising M30 trends indicate early apoptotic activity and help distinguish apoptosis from necrosis, particularly when interpreted alongside M65 (full-length keratin-18) and M30 ratios. Clinically, early increases in M30, especially when accompanied by elevations in miR-122 and GLDH, strongly predict progressive yet potentially reversible DILI and may warrant immediate therapeutic modification (Thulin et al., 2014; Church and Watkins, 2017; Korver et al., 2021). Again, these markers capture hepatocellular injury and vulnerability rather than CYP phenotype *per se*, but are mechanistically linked to the risk that any given genotype–phenotype mismatch will translate into clinically relevant harm.

Immunometabolic markers, including **soluble CD163** (sCD163), which reflects macrophage and Kupffer cell activation, and the IDO1-driven kynurenine/tryptophan ratio, indicative of immunomediated metabolic exhaustion and tolerance shift, are closely associated with inflammation-mediated CYP suppression and phenoconversion, particularly in critical illness and oncology (Kazankov et al., 2014; Li et al., 2025). These markers help differentiate primary hepatotoxicity from immune- or tumor-driven inflammation and may influence the selection of immunosuppressive or anti-inflammatory strategies.

#### 4.7.2 Integrated biomarkers matrix framework

**FIGURE 1:**
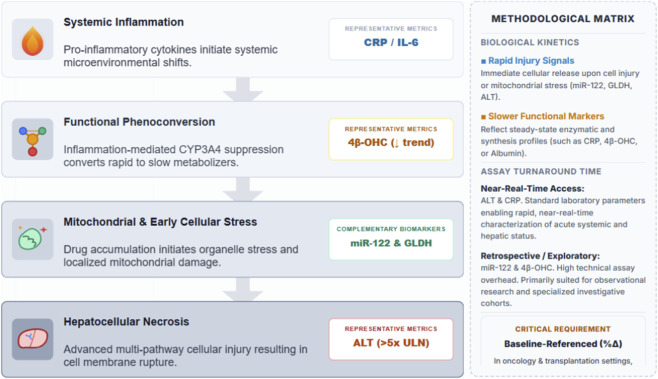
A theoretical framework delineates the cascade from systemic inflammation to functional phenoconversion and subsequent hepatocellular necrosis. The conceptual flowchart outlines a hypothesized, multi-level causal cascade in which specific pathophysiological triggers induce functional shifts in metabolic phenotype, ultimately resulting in structural cellular injury. In this top-to-bottom cascade, systemic inflammation, characterized by routine inflammatory proteins such as CRP and IL-6, serves as the upstream initiator of downstream inflammation-mediated CYP suppression. This suppression leads to functional phenoconversion, wherein a genotypically normal metabolizer transiently exhibits a slow-metabolizer phenotype. This metabolic alteration is theoretically indicated by the slow functional marker 4β-OHC. Investigation of this phase necessitates baseline-referenced designs, such as percent change, rather than static thresholds, due to the substantial baseline inter-individual heterogeneity observed in complex clinical contexts like oncology or transplantation. The subsequent hypothetical accumulation of toxic drugs provokes a progressive intracellular stress response, measured by two independent and complementary parameters: miR-122, which reflects early hepatocellular stress, and GLDH, which is specific for established mitochondrial injury. Although these tissue injury signals are biologically rapid, current assay turnaround times for miR-122 and 4β-OHC restrict their use to retrospective, exploratory research rather than acute, real-time clinical decision-making. The final stage, characterized by advanced multi-pathway cellular necrosis and parenchymal collapse, is indicated by elevated ALT levels. This schematic constitutes a non-validated, hypothesis-generating exploratory framework intended exclusively to conceptualize biological cross-talk and phenotype-genotype discordance during drug exposure. It is not intended for clinical risk classification, real-time patient triage, or active medical guidance (Fontana et al., 2023).

Each biomarker demonstrates unique kinetics, release mechanisms, and clearance rates. Therefore, mechanistic insights are best achieved through integrated, context-aware interpretation (Table 2; Figure 1). It is essential to distinguish between real-time indicators of acute cellular injury and slower functional or systemic metabolic markers. Consistent with current evidence, the biomarker matrix is now presented as a non-validated, hypothesis-generating framework rather than a prescriptive clinical algorithm. The proposed exploratory framework integrates real-time parameters, including rapid-response tissue injury markers such as miR-122, GLDH, and M30. The concurrent elevation of these markers is hypothesized to reflect multi-pathway cellular stress, although they lack established predictive value. These are considered alongside slower-evolving systemic and functional variables, such as systemic inflammatory status (via CRP/IL-6), eGFR, and albumin (Figure 1; Table 3) (Thulin et al., 2014; Shah and Smith, 2015c; Stanke-Labesque et al., 2020; Thakur et al., 2024).

**TABLE 2:** The proposed non-validated biomarker framework for monitoring and risk assessment of DILI.

Biomarker	Proposed signal threshold (exploratory)	Weight of evidence (0–2)	Rationale and literature basis	Hypothetical interpretations and future research directions
ALT level	>3x ULN	2	Standard safety signal warrants further investigation	Traditionally, it prompts the use of standard diagnostic protocols (e.g., AASLD/Hy’s law screening) in clinical trials to evaluate the underlying etiology
miR-122	>2x baseline or rising trend	1–2	Highly liver-specific; precedes ALT in some experimental models	Could hypothetically warrant more frequent sampling in clinical cohorts to investigate subclinical hepatocyte stress
CRP/IL-6	CRP >30 mg/L or IL-6 >20 pg/mL	2	Systemic inflammatory markers associated with downstream phenoconversion risk via CYP suppression	May theoretically signal a need to evaluate inflammation-mediated DDI or potential phenoconversion in exploratory settings
GLDH	Above ULN or rising trend	1–2	Specific for mitochondrial injury; non-muscle origin	Serves as a potential investigative marker to assess mitochondrial toxicity risk via serial measurements
sCD163/M30	Significant upward trend	1	Reflects macrophage activation or apoptosis	Serves as an exploratory follow-up endpoint to identify specific mechanistic pathways in clinical studies

The proposed framework stratifies based on their potential utility for detecting and monitoring DILI. This assessment relies on hypothetical signal thresholds and weight-of-evidence scores from preliminary literature. The framework combines established markers of hepatocellular damage with emerging indicators of mitochondrial injury, early cellular stress, and systemic inflammation. This integration aims to inform future clinical validation and mechanistic studies. By examining pathways such as macrophage activation and CYP3A4-mediated phenoconversion, the theoretical matrix supports risk-mitigation strategies and individualized drug-exposure assessments. Importantly, this matrix is intended solely as a hypothetical construct for research design and literature review. It is not a validated clinical decision-making tool. The concurrent elevation of miR-122, GLDH, and M30 should be interpreted as an exploratory marker of multi-pathway cellular stress rather than a metric with established predictive value.

**TABLE 3:** Proposed Research Framework for an Integrated Biomarker Index.

Biomarker	Primary domain/Intended use	Proposed signal threshold	Mechanistic response framework	Assay feasibility	References
ALT level	Hepatocellular injury/Safety flag	>3x ULN	Initiate comprehensive DILI assessment (AASLD/Hy’s law check); evaluate symptoms, bilirubin, and INR; consider drug interruption depending on context	Routine (widely available)	AASLD guidelines 2023; Hy’s law criteria
miR-122	Early hepatocyte stress/DILI risk	>2x baseline or rising trend	Increase monitoring frequency; evaluate for subclinical hepatocyte stress; review concomitant hepatotoxic drugs	Specialized (research use)	Thulin et al. (2014), Thulin et al. (2017); Antoine and Dear (2017); Zhang et al. (2024)
CRP/IL-6	Systemic inflammation induced CYP phenoconversion risk	CRP >30 mg/L or elevated IL-6	Review potential for CYP/transport-mediated DDIs; consider TDM and reassessment of high-risk drugs	Routine (CRP)/Specialized (IL-6)	Schmith and Foss (2008); Klomp et al. (2020); Yokota et al. (2025)
GLDH	Mitochondrial injury/Severity modifier	Above ULN or rising trend	Assess mitochondrial toxicity risk; consider serial measurement, especially with APAP or valproate	Specialized (limited)	Church et al. (2020); Aubrecht et al. (2025)
4β-OHC	CYP3A4 functional phenotype	>30% decrease from baseline	Evaluate CYP3A4 metabolic capacity; assess risk of over-exposure; use alongside drug levels	Research (MS-based)	Diczfalusy et al. (2009); Fahmi et al. (2008); Li et al. (2019)
sCD163/M30	Immune activation/Apoptosis	Significant upward trend	Exploratory follow-up to identify specific mechanistic pathways (immune-mediated vs. apoptotic)	Research (ELISA)	Kazankov et al. (2014); Korver et al. (2021); Thulin et al. (2014)

This table summarizes a proposed exploratory biomarker panel and signal thresholds to support hypothesis generation and the design of prospective validation studies. It is not a validated clinical decision tool.

**FIGURE 2:**
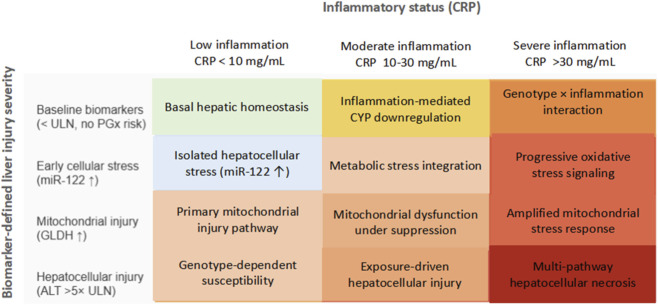
Conceptual matrix of hypothesized DILI and inflammation-driven phenoconversion risks. The matrix illustrates the synergistic effect between biomarker-defined liver injury severity (Y-axis) and systemic inflammatory status (X-axis, classified by CRP levels). Color-coded risk strata indicate a progressive transition from low PGx-adjusted risk (light green) to overt DILI and severe liver injury (dark red). The model highlights that moderate-to-severe inflammation acts as a critical driver of functional phenoconversion, primarily via inflammation-mediated CYP downregulation, which lowers the threshold for metabolic stress integration and substantially amplifies the potential for exposure-driven hepatocellular injury, even in genotype-predicted normal metabolizers. This graphic represents a non-validated, purely conceptual schematic of research hypotheses and is not intended for clinical triage, risk classification, or therapeutic decisions.

For example, during systemic inflammation characterized by elevated CRP (>30 mg/L) or IL-6 (>20 pg/mL), patients previously classified as normal metabolizers by genotype may gradually shift toward a slow-metabolizer status due to inflammation-mediated CYP suppression. A slower decrease in the metabolic probe 4β-OHC, driven by this inflammatory context and observed alongside rapid increases in acute injury markers (miR-122 and GLDH) and trends in immune activation (rising sCD163), may indicate a combined risk of phenoconversion and DILI (Figure 2). In investigative or clinical trial contexts, such biomarker patterns may justify exploratory therapeutic drug monitoring and broader metabolic profile assessments rather than immediate clinical intervention (Thakur et al., 2024; Church et al., 2025).

These scenarios support the hypothesis that overlapping but temporally distinct domains—dynamic metabolic phenotypes, real-time hepatocellular injury, and systemic immune activation—should be evaluated collectively to anticipate clinically meaningful genotype–phenotype discordance.

Methodological considerations of feasibility and context are essential for interpreting this exploratory framework. First, it is important to distinguish between independence and redundancy: miR-122 and GLDH are not interchangeable and offer complementary insights (Church et al., 2020; Aubrecht et al., 2025). The miR-122 identifies early hepatocellular stress, while GLDH is more specific for established mitochondrial injury (Thulin et al., 2014; Antoine and Dear, 2017). Second, both biological kinetics and assay turnaround times limit the current applicability of these parameters, requiring a clear distinction between rapid injury signals and slower metabolic indicators. Routine markers such as ALT and CRP enable near-real-time assessment of acute systemic and hepatic status. In contrast, although miR-122 rapidly indicates cellular damage, its current assay lead time, combined with the slower clearance and synthesis kinetics of functional markers such as 4β-OHC, makes these parameters unsuitable for retrospective or exploratory monitoring in clinical trials rather than acute, real-time clinical management (Schmith and Foss, 2008; Klomp et al., 2020; Yokota et al., 2025). Third, clinical heterogeneity reduces the utility of fixed universal cut-offs. In oncology and transplantation, changes from an individual’s baseline, such as percentage change in the slow functional marker 4β-OHC or the rapid injury marker miR-122, are likely more informative than absolute thresholds. Consequently, future validation studies should use baseline-referenced designs in these settings to characterize these distinct temporal axes (Fahmi et al., 2008; Diczfalusy et al., 2009; Zhang et al., 2024).

#### 4.7.3 Theoretical framework for biomarker integration

The exploratory biomarker integration concept offers a non-validated, hypothesis-generating framework to understand how intersecting pathophysiological domains, such as systemic inflammation and cell-specific injury, may alter the biological threshold for progressive parenchymal tissue damage. As shown in the conceptual matrix (Figure 2), drug exposure under varying microenvironmental conditions produces distinct pathomechanistic configurations. These illustrate how specific triggers induce functional shifts in metabolic phenotype that may lead to structural injury.

In a low-inflammation context (CRP <10 mg/L), biomarker alterations indicate localized, isolated structural responses. These progress from basal hepatic homeostasis to either an isolated hepatocellular stress signal (miR-122) or a primary mitochondrial injury pathway (GLDH), suggesting limited cellular involvement. In contrast, moderate-to-severe systemic inflammatory triggers are proposed to induce CYP downregulation via inflammation-mediated mechanisms. This transition modifies the metabolic phenotype, converting genotypically normal metabolizers into functional slow metabolizers and shifting the pathway toward metabolic stress integration and mitochondrial dysfunction.

The convergence of severe systemic inflammation (CRP >30 mg/L) and advanced biomarker-defined tissue injury (ALT > 5x ULN) constitutes a critical genotype-inflammation interaction and a progressive oxidative stress signaling pathway. In this subcellular environment, the cascade advances from an amplified mitochondrial stress response to catastrophic multi-pathway hepatocellular necrosis (Figure 2). Research consortia such as SAFE-T (Safer and Faster Evidence-based Translation) have shown that evaluating concurrent multi-parameter pathways, including parallel necrosis and apoptosis signals, characterizes tissue-damaging phenotypes with greater mechanistic fidelity than conventional standalone assays (Church and Watkins, 2017; Church et al., 2019; 2025). However, these specific multi-biomarker thresholds remain under exploratory validation by investigative networks such as the DILI (Hey-Hadavi et al., 2021).

This integrated matrix serves exclusively as a conceptual tool for evaluating phenotype-genotype discordance and assessing the biological plausibility of parenchymal collapse in drug safety evaluation. It is entirely separate from active clinical triage or immediate patient-management protocols.

#### 4.7.4 Kinetic relationships and the early-warning window

The proposed concept’s weighting is based on kinetic relationships observed in human cohorts and supported by preclinical models. These findings challenge the traditional diagnostic paradigm based on transaminases. Within 6–24 h after drug exposure, a rapid rise in circulating miR-122, followed by an early increase in GLDH, marks the earliest molecular signature of hepatocellular and mitochondrial stress (Antoine and Dear, 2017; Thulin et al., 2017). This period defines a critical subclinical early-warning window during which cellular injury is present, even though conventional laboratory abnormalities have not yet appeared. In contrast, ALT and AST typically rise only after 48–72 h or later and are less organ-specific. This delay creates a diagnostic lag and narrows the window for therapeutic intervention, potentially increasing the risk of overt necrosis (Lee et al., 2017; Wang et al., 2023). While these kinetic patterns are statistically robust at the group level, they are influenced by the toxicodynamic properties of the compound, such as direct hepatotoxicants versus idiosyncratic DILI mechanisms, and by individual metabolic and CYP capacity. Therefore, in high-risk clinical scenarios, early targeted sampling of miR-122 and GLDH, combined with serial measurements over the first 72 h, offers a promising strategy for future studies to detect subclinical DILI and emerging phenotype shifts.

#### 4.7.5 Illustrative clinical scenario: Inflammation, transporter genotype and functional phenoconversion

A representative clinical scenario demonstrates the interplay between genotype, inflammation, and transporter status. A patient without known loss-of-function CYP3A4 variants (e.g., CYP3A4 *1/*1) may show preserved CYP3A4 activity under stable conditions. However, unlike *CYP2D6* or *CYP2C19*, this genotype does not constitute a robust stand-alone actionable pharmacogenomic phenotype (Fahmi et al., 2008, p. 2; Dutreix et al., 2014, p. 2; de Jong et al., 2023). During severe infection or an inflammatory bowel disease flare, significant elevations in CRP and IL-6 can suppress CYP3A4 expression and activity. Consequently, such a patient may functionally resemble a poor metabolizer despite having a “low-risk” genotype (Lenoir et al., 2021; Benzi et al., 2023). Maintaining standard dosing in this context can substantially increase drug exposure and lead to hepatotoxicity or bone marrow suppression, outcomes that germline genotyping alone cannot predict.

Drug disposition becomes more complex when hepatic transporter variants are present. Polymorphisms in SLCO1B*1 or *ABCC2* (e.g., SLCO1B1*5, *ABCC2* c.-24C>T) reduce hepatic uptake or biliary excretion, limiting drug clearance (Simon et al., 2013; Roszkiewicz et al., 2021). During systemic inflammation, shared cytokine pathways downregulate both CYP enzymes and transporters. This results in a dual bottleneck with reduced hepatic uptake or excretion and diminished metabolic capacity. In this context, a patient with an *SLCO1B1* risk allele and IL-6-mediated CYP3A4 suppression may experience significantly increased exposure to statins, MTX, or other CYP3A4/OATP1B1 substrates, even when the pharmacogenomic profile appears favorable (Fahmi et al., 2008; Shah and Smith, 2015b; Roszkiewicz et al., 2021; Molnár et al., 2026).

Within this hypothesis-generating framework, crossing exploratory biomarker signal zones (e.g., elevated CRP or IL-6, decreased 4β-OHC, increased miR-122 or GLDH) should not automatically trigger specific interventions. Instead, these findings should prompt a more intensive clinical review. This includes reassessment of high-risk agents, consideration of dose adjustment or temporary interruption in accordance with established guidelines, and targeted management of the underlying inflammatory process. Integrating CYP and transporter genotypes with dynamic biomarkers such as miR-122, GLDH, 4β-OHC, CRP, and IL-6 may allow detection of subclinical liver stress and emerging genotype-phenotype discrepancies and their consequences, particularly in patients with polypharmacy, systemic inflammation, or unstable organ function. Since no single parameter is sufficiently sensitive or specific in all scenarios, effective implementation of this approach will require multivariate, algorithmic interpretation of trends and baseline-referenced changes rather than reliance on isolated values.

### 4.8 Clinical advantages of an indexed biomarker matrix framework in PGx-Guided therapy

An integrated, index-based biomarker matrix approach could aid in expanding PGx-guided therapies by combining mechanistic markers within a patient-group-specific precision-monitoring strategy. Conceptually, this framework is most relevant for patients at high pharmacokinetic risk—those receiving narrow-therapeutic-index drugs, prodrugs, or CYP/P-gp substrates; individuals with polypharmacy; and patients with acute or chronic inflammatory conditions (infection, autoimmune disease, malignancy) or unstable organ function, in whom divergence between genotype-predicted and observed metabolic phenotype is most likely (Jeong, 2001; Yokota et al., 2025).

Within this hypothesis-generating framework, the integrated analysis of routine (CRP, transaminases), functional (4β-OHC), and molecular (miR-122, GLDH, M30, sCD163) biomarkers offers a novel strategy to interpret clinically significant genotype–phenotype discordance. This approach reliably shows the functional consequences of altered CYP activity compared to genetic predictions. Combining these multi-level parameters systematically reflects the main mechanistic domains: systemic inflammation and risk of phenoconversion (CRP, IL-6), mitochondrial and oxidative stress (AST/ALT ratio, GLDH), early hepatocyte stress and apoptosis (miR-122, M30), and actual CYP3A4 metabolic capacity (4β-OHC) (Thulin et al., 2017; Zhang et al., 2024). For example, CRP values above ∼30 mg/L or clearly elevated IL-6 levels indicate a high inflammatory burden and an increased likelihood of inflammation-mediated phenoconversion, warranting closer therapeutic drug monitoring of CYP-metabolized drugs.

An increase in the CRP/IL-6, miR-122, GLDH, or De Ritis ratio in the hypothetical model presented could facilitate more frequent monitoring, structured reassessment of dose, and drug selection. Ultimately, for this approach to be clinically applicable, the hypothetical biomarker matrix could be linked to an algorithm that can be integrated into clinical applications and incorporated into decision support systems. However, it is important to emphasize that all of this needs to be tested prospectively to see whether the components of the hypothetical matrix can actually improve the prediction of exposure, DILI, and drug response in specific patient populations.

## 5 Discussion

Pharmacogenetic information, particularly germline CYP and transporter allelic variants, alone cannot fully predict an individual’s metabolic phenotype. Evidence from several clinical areas—transplantation, oncology, and critical illness—shows that inflammation, organ damage, polypharmacy, redox and mitochondrial stress, and transporter dysregulation, individually and in combination, can cause significant differences between genotype-predicted and observed drug use. The first hypothesis investigated the mechanistic pathways underlying this discrepancy. The literature supports a multifactorial model. Cytokine cascades (IL-6/STAT3, NF-κB, MAPK), nuclear receptor repression (PXR, CAR), altered mRNA stability, ubiquitin-proteasome-mediated degradation, mechanism-based enzyme inactivation, and transporter modulation can all contribute to changes in functional CYP and transporter activity. These effects are isoform- and context-specific. These subcellular effects and interactions cannot be interpreted as a single, direct linear pathway, but rather as a combination of mechanisms at different levels and with varying weights across different diseases and treatment regimens. The second hypothesis examined which biomarkers are mechanistically justified and supported by new evidence as biomarker candidates. The panel considered CRP/IL-6, 4β-OHC, miR-122, GLDH, M30, and sCD163. These biomarkers span distinct but complementary domains: systemic inflammation and inflammation-induced phenoconversion risk (CRP, IL-6); CYP3A4 functional capacity (4β-OHC); early hepatocyte stress and apoptosis (miR-122, M30); and mitochondrial injury and macrophage activation (GLDH, sCD163). Each marker has shown a signal in human studies or translational models relevant to DILI or altered drug exposure, but none have been validated as a unified score. The data support the plausibility that combining these mechanistically orthogonal domains could improve early detection of phenotype shifts and emerging liver injury compared to single-marker, Transaminase-centered approaches. This potential is particularly relevant in high-risk settings such as polypharmacy, malignancy, transplantation, or severe chronic and autoimmune inflammation.

The third hypothesis proposed integrating these biomarkers into a weighted Precision Monitoring framework to inform adaptive, context-dependent drug dosing in future studies. Conceptually, this biomarker framework offers a structured approach to linking mechanistic domains to potential monitoring responses, such as deciding when to intensify therapeutic drug monitoring, assess DDI, or re-evaluate high-risk medications. However, this framework remains exploratory and has not been validated across diverse populations, drugs, or clinical settings. It is not yet clear whether a composite score or individual markers best predict drug exposure, DILI, or clinical outcomes. The degree of independence versus redundancy among markers also remains unknown. Feasibility is currently limited by assay availability, cost, and turnaround time. Currently, miR-122, GLDH (by specialized assays), M30, sCD163, and 4β-OHC are mainly restricted to research or specialist laboratories.

The review is a mechanistically informed summary that provides a synthesis aimed at guiding future research and development. Several key steps are recommended: (i) conducting condition- and drug-specific prospective studies to determine whether integrating select biomarkers with PGx and standard laboratory tests enhances the prediction of drug exposure and DILI risk; (ii) systematically evaluating optimal cut-offs, biomarker combinations, and sampling timing; (iii) rigorously assessing assay standardization, accessibility, and health-economic implications. The clinical application of the conceptual proposal is possible only if these studies demonstrate consistent clinical benefit, cost-effectiveness, and operational feasibility. Within these acknowledged limitations, this review provides a comprehensive synthesis and actionable framework for advancing PGx from a static, genotype-centric approach to a dynamic, biomarker-informed paradigm that explicitly incorporates inflammation, redox state, transporter function, and hepatocellular resilience as key modifiers of real-world drug response.

## 6 Conclusion

To increase the effectiveness of PGx-based drug therapy, it is necessary to integrate it into a dynamic framework that reflects the complexity and variability of real-world drug response. Although genotype provides a stable basis for understanding pharmacokinetic predisposition, metabolic phenotype is influenced by factors such as inflammation, organ function, polypharmacy, redox balance, and transporter activity. This analysis shows that combining routine clinical biomarkers (including CRP and transaminases) with functional (4β-OHC) and molecular markers (miR-122, GLDH, M30, sCD163) can serve as a systematic approach to resolve genotype-phenotype discrepancies and identify emerging risk of DILI.

However, these biomarkers should currently be considered promising candidates for prospective validation rather than parts of established clinical algorithms. Realizing their full potential requires rigorous, context-specific studies to define clinically meaningful thresholds, optimal sampling strategies, and the true incremental value of each marker. It also requires addressing assay standardization, accessibility, and cost-effectiveness. Only through such systematic evaluation can it be ensured that biomarker matrices provide real-world benefit and are feasible for clinical adoption.

If future evidence confirms that this approach improves the prediction of drug exposure, toxicity, and clinical outcomes, broader implementation and incorporation in therapeutic guidelines may be warranted. Until such evidence is available, the proposed framework may serve as a hypothetical basis for the design, ranking, and interpretation of critical validation studies, and may point to a rethinking and expansion of PGx as a dynamic, context-aware discipline at the forefront of precision medicine.

## 7 Future perspectives

Moving beyond static, genotype-centric models is the next essential Frontier in translational pharmacology and drug safety evaluation. The conceptual framework in this review shows that real-world drug response is dynamic, shaped by a non-linear network of inflammation-mediated enzyme suppression, oxidative stress, and altered cellular resilience. Future research must prioritize capturing this functional phenotype-genotype discordance through functional phenoconversion and systems biology. Advancing this field requires a shift from isolated biomarker assessments to a comprehensive, multi-omic paradigm. Integrating genome-wide PGx profiles with longitudinal transcriptomic, proteomic, and metabolomic data from diverse patient cohorts is critical to deciphering the context-specific, multi-layered mechanisms driving transient changes in metabolic capacity.

## Glossary

4β-OHC: β-hydroxycholesterol
ABCB1: ATP-binding cassette sub-family B member 1 (P-glycoprotein)
ABCC2: ATP-binding cassette sub-family C member 2 (MRP2)
ABCG2: ATP-binding cassette sub-family G member two
ALP: alkaline phosphatase
ALT: alanine aminotransferase
ANC: absolute neutrophil count
AST: aspartate aminotransferase
BSEP: bile salt export pump
CAR: constitutive androstane receptor
CI: confidence interval
**C**_**max**_: maximum plasma concentration
CYP: cytochrome P450 (enzyme family)
DILI: drug-induced liver injury
DNMT: DNA methyltransferase
eGFR: estimated glomerular filtration rate
GCLC: glutamate-cysteine ligase catalytic subunit
GCLM: glutamate-cysteine ligase modifier subunit
GGT (γ-GT): gamma-glutamyl-transferase
GSH: reduced glutathione
GSSG: oxidized glutathione
GLDH: glutamate dehydrogenase
HLA: human leukocyte antigen
IC50: half-maximal inhibitory concentration
IDO1: indoleamine 2,3-dioxygenase one
IL-1β: interleukin-1 beta
IL-6: interleukin-6
Km: Michaelis–Menten constant
**k**_**inact**_: maximal inactivation rate constant (mechanism-based inactivation)
**K**_**i**_: inhibition constant
mPTP: mitochondrial permeability transition pore
M30: caspase-cleaved keratin-18 fragment (apoptosis marker)
miR-122: microRNA-122
MRP2: multidrug resistance-associated protein 2 (ABCC2)
Nrf2: nuclear factor erythroid 2-related factor 2
OATP: organic anion transporting polypeptide (OATP1B1/1B3)
P-gp: P-glycoprotein (ABCB1)
PDR: probe drug response
PXR: pregnane X receptor
PK: pharmacokinetics
PD: pharmacodynamics
PGx: pharmacogenomics
qPCR: quantitative polymerase chain reaction
ROS/RNS: reactive oxygen/nitrogen species
RT-qPCR: reverse transcription quantitative PCR
sCD163: soluble CD163 (macrophage activation marker)
SLCO1B1: solute carrier organic anion transporter family member 1B1 (OATP1B1)
STAT3: signal transducer and activator of transcription three
TDM: therapeutic drug monitoring
TNFα: tumor necrosis factor alpha
ULN: upper limit of normal
