# Supplementary material for: Dynamic phenotype monitoring to prevent genotype–phenotype discrepancies in pharmacogenetic-guided drug therapy
Source: Front Pharmacol. 2026 Jul 2;17:1867629. doi: 10.3389/fphar.2026.1867629 (PMC13372700; doi:10.3389/fphar.2026.1867629)

**Supplementary Materials 1:**

("pharmacogenomics"[MeSH Terms] OR "pharmacogenomics"[Title/Abstract] OR "PGx"[Title/Abstract]) AND

("phenoconversion"[Title/Abstract] OR "phenotype conversion"[Title/Abstract]) AND

("cytochrome P450"[MeSH Terms] OR "CYP"[Title/Abstract] OR "CYP3A4"[Title/Abstract]) AND

("inflammation"[MeSH Terms] OR "IL-6"[Title/Abstract] OR "interleukin-6"[MeSH Terms]) AND

("biomarkers"[MeSH Terms] OR "miR-122"[Title/Abstract] OR "4β-hydroxycholesterol"[Title/Abstract] OR "GLDH"[Title/Abstract] OR "M30"[Title/Abstract]) AND

("drug-induced liver injury"[MeSH Terms] OR "DILI"[Title/Abstract]) AND

("transporters"[Title/Abstract] OR "SLCO1B1"[Title/Abstract] OR "ABCB1"[Title/Abstract] OR "ABCC2"[Title/Abstract]) AND

("therapeutic drug monitoring"[MeSH Terms] OR "TDM"[Title/Abstract])

Filters: Publication date from 2000/01/01 to 2026/03/31; English


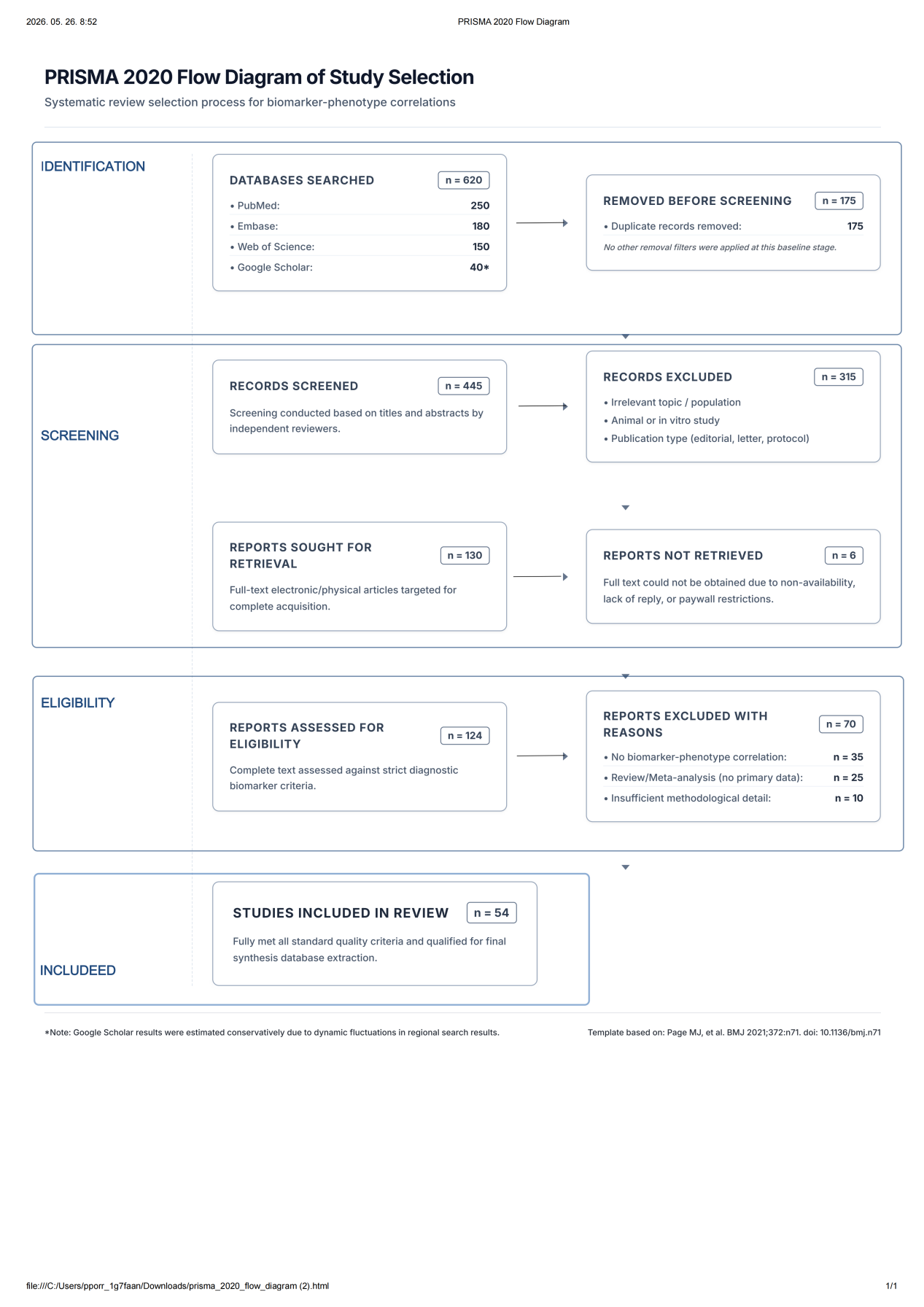

Supplement: Supplementary file 1 [file Supplementaryfile1.docx]
